# *Toxoplasma gondii* CDPK3 Controls the Intracellular Proliferation of Parasites in Macrophages

**DOI:** 10.3389/fimmu.2022.905142

**Published:** 2022-06-10

**Authors:** Minmin Wu, Ran An, Nan Zhou, Ying Chen, Haijian Cai, Qi Yan, Ru Wang, Qingli Luo, Li Yu, Lijian Chen, Jian Du

**Affiliations:** ^1^ Department of Biochemistry and Molecular Biology, School of Basic Medical Sciences, Anhui Medical University, Hefei, China; ^2^ The Research Center for Infectious Diseases, School of Basic Medical Sciences, Anhui Medical University, Hefei, China; ^3^ The Provincial Key Laboratory of Zoonoses of High Institutions of Anhui, Anhui Medical University, Hefei, China; ^4^ The Key Laboratory of Microbiology and Parasitology of Anhui Province, Anhui Medical University, Hefei, China; ^5^ School of Nursing, Anhui Medical University, Hefei, China; ^6^ Department of Anesthesiology, The First Affiliated Hospital of Anhui Medical University, Hefei, China

**Keywords:** *Toxoplasma gondii*, CDPK3, IRGs, GBPs, parasitophorous vacuole

## Abstract

Interferon-γ (IFN-γ)-activated macrophages restrain the replication of intracellular parasites and disrupt the integrity of vacuolar pathogens. The growth of the less virulent type II strain of *Toxoplasma gondii* (such as ME49) was strongly inhibited by IFN-γ-activated murine macrophages. However, the mechanism of resistance is poorly understood. Immunity-related GTPases (IRGs) as well as guanylate-binding proteins (GBPs) contributed to this antiparasitic effect. Previous studies showed the cassette of autophagy-related proteins including Atg7, Atg3, and Atg12-Atg5-Atg16L1 complex, plays crucial roles in the proper targeting of IFN-γ effectors onto the parasitophorous vacuole (PV) membrane of *Toxoplasma gondii* and subsequent control of parasites. *Tg*CDPK3 is a calcium dependent protein kinase, located on the parasite periphery, plays a crucial role in parasite egress. Herein, we show that the less virulent strain CDPK3 (ME49, type II) can enhance autophagy activation and interacts with host autophagy proteins Atg3 and Atg5. Infection with CDPK3-deficient ME49 strain resulted in decreased localization of IRGs and GBPs around PV membrane. *In vitro* proliferation and plaque assays showed that CDPK3-deficient ME49 strain replicated significantly more quickly than wild-type parasites. These data suggested that *Tg*CDPK3 interacts with the host Atg3 and Atg5 to promote the localization of IRGs and GBPs around PV membrane and inhibits the intracellular proliferation of parasites, which is beneficial to the less virulent strain of *Toxoplasma gondii* long-term latency in host cells.

## Introduction

*Toxoplasma gondii* is one of the most successful protozoan parasites owing to its ability to manipulate the immune system and establish a chronic infection. Humans are mainly infected with *T. gondii* through ingestion of undercooked meat containing tissue cysts or ingestion of food and water contaminated with oocysts excreted by cats (1, 2). Toxoplasmosis has variable outcomes in the host. Immunocompetent individuals infected with *T. gondii* are often asymptomatic or develop mild symptoms. However, it may cause serious illness and death in individuals with immune deficiencies and the developing fetus of pregnant women (1, 3). Types I, II and III of *T. gondii* are the classical North American and European strains. Type I strains are mainly RH and GT1, type II strains are PRU and ME49, and type III strains are mainly CEP, and their virulence varies greatly. Type I is high virulent, type II is intermediate virulent, and type III is avirulent (4, 5).. Unlike in North America and Europe, *T. gondii* strains in South American are genetically more diverse. Our previous studies have demonstrated that Chinese 1(ToxoDB#9) lineage is the most common genotype in East Asia, especially in China (> 79% based on 60 isolates) (6–8). Moreover, our group have found that the isolates of Chinese 1 vary in their virulence in mice; among these, WH3 exhibits high virulence, whereas WH6 exhibits low virulence (8–11). Different *T. gondii* strains vary in their genomes, resulting in divergent resistance to these host defense mechanisms. For example, most strains of *T. gondii* can survive within naïve macrophages, while macrophages acquire the ability to kill or inhibit parasites when activated by exposure to Interferon-γ (IFN-γ). Not all strains of *T. gondii* are susceptible to clearance in IFN-γ-activated macrophages. The virulent type I strain resists immune-modulating cargo recruitment and consequently avoid clearance, while intermediate virulent type II and avirulent type III parasites are unable to block immune-modulating cargo recruitment and are destroyed (12, 13).

*Toxoplasma gondii* proliferates within a special endocytic vacuole, called the parasitophorous vacuole (PV). IFN-γ is the main cytokine involved in activating the cellular autonomous immune response to control the proliferation of parasites within the PV (14–16). In murine cells, IFN-γ upregulates immune-related GTPase, such as p47 IFN-γ–regulated GTPases (IRGs) and p65 guanylate binding protein (GBPs), which play an important role in host defense. These effectors quickly localize on and around the PV membrane, resulting in the destruction of PV membrane and subsequent parasite death (17, 18). The autophagy-related proteins Atg7, Atg3 and the Atg12-Atg5-Atg16L1 complex, which are involved in delivery and conjugation of the ubiquitin-like protein microtubule-associated protein 1 light chain 3 (LC3) to the autophagosomal membrane, are necessary to target the IRGs and GBPs to the PV membrane (19, 20). IFN-γ-inducible immune-related GTPase mediated host defense through autophagy proteins plays important roles in the control of *T. gondii* infection. Furthermore, CD40 interacts with CD40L (CD154) expressed on the surface of T cells is also through an autophagy-dependent pathway to trigger the killing of the parasite (21, 22). *T. gondii* calcium-dependent kinase 3 is localized to the periphery of the parasite, and plays a key role in the rapid egress from host cells (23). Herein, to explore the role of *Tg*CDPK3 in the less virulent strain, we constructed ME49*Δcdpk3* strain through CRISPR/Cas9 technology. Using wild type ME49 strain and ME49*Δcdpk3* strain to infect murine macrophages, we found that *Tg*CDPK3 interacted with autophagy-related proteins Atg5 and Atg3, then induced autophagy activation, which promoted recruitment of IRGs and GBPs to PV membrane. Unlike their virulent strains, the interaction of avirulent or less virulent *T. gondii* strains with macrophage and dendritic cell initiates phagocytosis and subsequent active penetration from within the phagosome to form a parasitophorous vacuole (24, 25). During this process, the surface effectors of the parasite may have the chance to hijack the host signal pathway. Our study demonstrated for the first time that CDPK3 of less virulent strain interacts with the host Atg3 and Atg5 to activate the host autophagy, and then leads to sequential recruitment of immune-related proteins to PV membrane, which promotes the control of acute infection and establishs long-term latency in the macrophages.

## Materials and Methods

### Ethical Statement

Six to eight weeks old female C57BL/6 mice were purchased from Anhui Medical University Laboratory Animal Center. All animals were maintained under standard conditions according to Chinese National Institute of Health Guide for the Care and Use of Experimental Animals. The animal experiments were approved by the Institutional Review Board of the Institute of Biomedicine at Anhui Medical University (permit number: LLSC20200036).

### Cell Culture

The HFF(SCRC-1041), HEK293T cell lines (CRL-3216) were purchased from the American Type Culture Collection. RAW264.7(CL-0190) cells were kindly provided by Stem Cell Bank, Chinese Academy of Sciences. Cell were cultured in DMEM (Biological Industries, Israel) supplemented with 10% FBS (Biological Industries, Israel) and 1% penicillin/streptomycin (Biological Industries, Israel).C57BL/6 embryonic fibroblasts (MEFs) were prepared from mice at day 14 post coitum and cultured in DMEM (Biological Industries, Israel) supplemented with 10% FBS (Biological Industries, Israel) and 1% penicillin/streptomycin (Biological Industries, Israel).

### Parasite Strains

*Toxoplasma gondii* type I strain RH and type II strain ME49 were propagated in human foreskin fibroblast (HFF, SCRC-1041). Chinese 1 strain WH3 and WH6 were previously isolated from animals and human in China and propagated in HFF cells.

### Plasmids, SiRNA and Reagents

Amplification of the open reading frame encoding *Tg*CDPK3 (GenBank ID: XM_002370317.2) was achieved through RT-PCR of the ME49 tachyzoite RNA. Then the full-length *Tg*CDPK3 was subcloned into the pGEX6P-1 vector (GE Healthcare, Wisconsin, USA), pEGFP-C2 (BD Biosciences) and pCMV-3Tag-2A vector (BD Bioscience). Atg5-MYC (#24922) and Atg7-MYC (#24921) were purchased from Addgene. Amplification of the full open reading frame encoding human Atg3 (GenBank ID: NM_022488.4) was achieved through reverse transcriptase polymerase chain reaction (PCR) of the whole mRNA of Hela cells. Then the full-length human Atg3 was subcloned into the pCMV-3Tag-2A vector (BD Bioscience). All constructs were subject to sequencing for verification. The pEGFP-MAP1LC3B plasmid was a kind gift from Professor Feng Li Jie at Anhui Medical University (26). Cells were cultured to reach between 60-70% confluency at the time of transfection. Plasmid transfection was performed using Lipofectamine 3000 transfection reagent (Thermo Fisher Scientific, China) in 12-well plates following the manufacturer’s instructions. RAW264.7 cells were transiently transfected with siRNA against Atg3 and Atg5 using Lipofectamine 3000 transfection reagent (Thermo Fisher Scientific, China) in 12-well plates following the manufacturer’s instructions. Atg3-siRNA^#1^: GGAAAGCAAGGACAGTATA (sense); Atg3-siRNA^#2^: GAAAGTGAAGGCATATCTT (sense); Atg3-siRNA^#3^: GTACATCACTTACGACAAA (sense); Atg5-siRNA^#1^: CCATCA ACCGGAAACTCAT (sense), Atg5-siRNA^#2^: GGAACATCACAGTACATTT, Atg5-siRNA^#3^: GCATCTGAGCTACCCAGAT were purchased from RiboBio Co., Ltd. (Guangzhou,China) and used for knockdo wn of Atg3 and Atg5, respectively. Nonsense siRNA was used as the negative control. 3-maleimidobenzoic acid (3-MA, 5mM,5142-23-4, Sigma-Aldrich, USA), mouse IFN-γ (100 U/ml,315-05, PeproTech, NJ, USA).

### Generation of CDPK3 -Deficient Strain of Type II ME49

All the primers and plasmids used in this study are listed in **Supplementary Table 1**. CDPK3-specific CRISPR plasmids were generated by replacing the UPRT targeting guide RNA in pSAG1:CAS9-U6:sgUPRT (Addgene plasmid #54467) with corresponding guide RNAs, using Q5 mutagenesis kit (New England Biolabs, Ipswich, MA, USA).the 5′-homology and 3′-homology arms of CDPK3 were amplified from the genomic DNA of type II ME49 strain, as well as the DHFR sequence amplified from pUPRT-DHFR-TS. Subsequently, the homologous arms of CDPK3 along with the selectable markers DHFR*-Ts were cloned into pUC19 using ClonExpress MultiS One Step Cloning Kit (Vazyme Biotech, Nanjing, China). Subsequently, corresponding gene-specific CRISPR plasmid and donor DNA fragments were electroporated into purified ME49 tachyzoites, selected with 1 μM pyrimethamine. A single clone through limiting dilution were seeded in 96-well plate with HFF cells. Positive clones were detected by PCR and Western blotting. Diagnostic PCRs (PCR1, PCR2, and PCR3) were used to identify individual clone using primers listed in **Supplementary Table 1**.

### Western Blot Analysis

RAW264.7 cells were harvested 24 hr after plasmid transfection or ME49*wt* parasites or ME49*Δcdpk3* parasites infection (MOI=3). The cells proteins were extracted using RIPA buffer (Beyotime, China) supplemented with protease inhibitor phenylmethylsulfonyl fluoride (1 mM, Beyotime, China). The lysate was separated on a 12% SDS-PAGE gel, and then proteins of different molecular weights were transferred to a nitrocellulose membrane (Millipore, USA). The membranes were blocked with 5% skimmed milk in TBST for 1.5 h at room temperature and then incubated at 4°C overnight with primary antibody of mouse anti-IRG-47 antibody (sc-390264;Santa Cruz, USA,1:1000), mouse anti-GBP1-5 antibody (sc-166960; Santa Cruz, USA,1:1000), rabbit anti-LC3II antibody (NB100-2220; Novus,USA, 1:1000), rabbit anti -GFP antibody (2956s;CST,USA1:2000), mouse anti-Myc Tag antibody (05-724;Millipore,USA,1:2000), mouse anti-GAPDH antibody (60004-1-lg;proteintech,China, 1:5000), rabbit anti -Beclin1 antibody (#3495; CST, USA,1:1000), mouse anti-p62 antibody (ab56416; Abcam, U.K,1:1000), rabbit anti -Atg5 antibody (10181-2-AP; proteintech, China, 1:1000), rabbit anti-Atg3 antibody (11262-2-AP; proteintech, China, 1:1000), mouse anti-Atg7 antibody (sc-376212; Santa Cruz, USA, 1:1000). *Tg*CDPK3 antibody was prepared by Taopu company (Taopu, shanghai, 1:1000). *T. gondii* actin antibody and profilin antibody were kindly provided by Professor Yu Li (Anhui Medical University, China, 1:2000). The membranes were incubated with the corresponding HRP-conjugated secondary antibodies (proteintech, China, 1:10000) for 1 h. Immunoreactivity was detected with ECL blot detection system (Bio-Rad, Hercules, USA). Optical density for each band was analyzed using Image J software. The results were normalized to GAPDH expression.

### GST Pull-Down Assay *In Vitro*

The purified soluble GST-CDPK3 or GS​​T was combined with Glutathione-Sepharose 4B beads (GE Healthcare, Germany). 293T cells lysate expressing Atg3-myc, Atg5-myc or Atg7-myc were incubated with GST-CDPK3-conjugated or GST-conjugated beads at 4°C for 2 hours, respectively. Subsequently, the conjugated beads were washed 3 times with pre-cooled PBS containing 1% Triton X-100, and washed 3 times with PBS. The pellet was fixed in Laemmli loading buffer and analyzed using western blot.

### Immunoprecipitation Assay

HEK293T cells co-transfected with the corresponding plasmids were lysed in lysis buffer (50 mM HEPES, pH 7.4, 150 mM NaCl, 2 mM EGTA and 1% Triton X-100) containing Complete™ protease inhibitor (Roche Applied Science, Indianapolis, USA). RAW264.7 cells infected with ME49*wt* parasites or ME49*Δcdpk3* parasites were lysed in a lysis buffer (50 mM HEPES, pH 7.4, 150 mM NaCl, 2 mM EGTA and 1% Triton X-100) containing Complete™ protease inhibitors (Roche Applied Science, Indianapolis, USA). The supernatants were collected and incubated with Protein A/G plus-agarose (Pierce) binds the corresponding antibody for 4 h at 4°C. Subsequently, the immunoprecipitants were washed 3 times with pre-cooled 0.1% Triton X-100 in lysis buffer, and then washed 3 times with PBS. The bound proteins were analyzed western blot.

### Immunofluorescence Assays

Macrophages or MEF cells were plated onto glass coverslips. Subsequently, the cells were fixed with 4% paraformaldehyde, permeabilized with 0.1% Triton X-100 in PBS, and blocked using 10% bovine serum albumin. The coverslips were incubated with rabbit anti -LC3II antibody (NB100-2220; Novus, USA), rabbit anti-GFP antibody (2956s; CST, USA), Anti-Myc Tag antibody (05-724; Millipore, USA), *Tg*CDPK3 antibody (taopu, shanghai) at 4°C overnight. followed by incubation with secondary antibodies conjugated to Rhodamine-conjugated goat anti-mouse IgG, Rhodamine-conjugated goat anti-rabbit IgG, FITC-conjugated goat anti-mouse IgG, FITC-conjugated goat anti-rabbit IgG for 1 h at 37°CC in the dark. DAPI dye were used for DNA visualization. Finally, the immunostained cells was analyzed using confocal laser microscopy (LSM880+airyscan, ZEISS, German) in three replicates from three biological experiments. The fluorescence intensity was analyzed using software ImageJ (v1.53c, National Institutes of Health, Bethesda, MD, USA).

### Immunity-Related GTPase Vacuole Recruitment

Macrophages or MEF cells pre-treated with 100 U/ml murine recombinant IFN-γ (PeproTech, NJ, USA) for 24 h, and were then infected with ME49*wt* parasites or ME49*Δcdpk3* parasites (MOI=3) for 4-6 h. Cells were fixed in PBS containing 4% paraformaldehyde for 15 min at room temperature, permeabilized with 0.1% Triton X-100 in PBS, and then blocked with 10% bovine serum albumin for 1 h at room temperature. Subsequently, *Tg*CDPK3 antibody (taopu, shanghai) was used to stain the *Toxoplasma gondii*, and immunity-related GTPase localization was determined by staining mouse anti-IRG-47 antibody (sc-390264; Santa Cruz, USA), mouse anti-GBP1-5 antibody (sc-166960; Santa Cruz, USA), rabbit anti -LC3II antibody (NB100-2220;Novus,USA),followed by incubation with secondary antibodies conjugated to Rhodamine-conjugated goat anti-mouse IgG, Rhodamine-conjugated goat anti-rabbit IgG, FITC-conjugated goat anti-mouse IgG,FITC-conjugated goat anti-rabbit IgG for 1 h at 37°C in the dark. DAPI dye was used for DNA visualization. Immunostained cells were then analyzed using confocal laser microscopy (LSM880+airyscan, ZEISS, German) in three replicates from three biological experiments. The number of IRG-47 or GBP1-5-containing positive vacuoles was determined by counting 10 fields at a magnification of 63× from three separate coverslips of three replicate experiments.

### Transmission EM

RAW264.7 cells were pre-treated with 100 U/ml mouse IFN-γ (PeproTech, NJ, USA), 0.1 ng/ml LPS (Sigma-Aldrich, USA) for 24 h were infected with ME49*wt* parasites or ME49*Δcdpk3* parasites (MOI=3) for 4~6 h. Cells were fixed with 2.5% glutaraldehyde in 0.1 M phosphate buffer at 4°C overnight. After washing with phosphate buffer, the cells were fixed in phosphate buffer with 1% OsO4 at 4°C for 2 hours and rinsed thoroughly with ddH_2_O. The 2% aqueous uranyl acetate was used for en bloc staining for 2 hours. and then the cells were dehydrated in a graded series of ethanol and embedded in Quetol 812 (Nissin EM). Silver sections were cut with an ultramicrotome, stained with lead citrate and uranyl acetate and observed with an electron microscope (Talos L120C G2, Thermo Scientific, USA).

### Quantitative Real-Time PCR

Total RNA was extracted, and sample RNA was reverse transcribed to cDNA using the PrimeScript™ RT Kit (TaKaRa, Dalian, China). Real-time qPCR was performed with the SYBR^®^ Premix Ex TaqTM II Kit (TaKaRa, Dalian, China). GAPDH and β-actin is used as a relative quantification control to evaluate transcript abundance. The expression levels of the following genes are examined: LC3, Beclin1, p62, *Tg*CDPK3, *Tg*SAG1. Each sample was measured in technical triplicates. and the data represent three separate experiments. Reactions were run on the Roche LC480II system. The primer sequences are listed in **Supplementary Table 2**.

### Replication Assay

1×10^5^ ME49*wt* parasites or ME49*Δcdpk3* parasites were used to infect MEF monolayer seeded on coverslips for 1 hours in a 12 well plate. Cells were washed twice with PBS and resuspended in normal culture medium. 24 hours after parasite infection, cells were fixed in 4% paraformaldehyde and stained for Giemsa staining. The number of parasites each parasitophorous vacuole (PV) (i.e., one, two, four, eight) was obtained by counting ≥100 PVs using a LEICA ICC50W microscope at 1000×magnification. The infected cells were processed for immunoblot and qPCR as previously described. All strains were tested 3 times or more independently, each with three internal replicates.

### Invasion Assay

To compare the invasion efficiency of the two strains, 1 × 10^5^ ME49*wt* parasites or ME49*Δcdpk3* parasites were allowed to invade confluent MEFs for 2 hours in a 24 well plate. Wells were then washed 3 times in PBS and fixed in 4% paraformaldehyde, and Rhodamine-conjugated anti-SAG1 antibody (GTX38936; GeneTex, USA) was used to detect non-invasive extracellular parasites. Following permeabilization with 0.2% Triton X-100 in PBS, all parasites were visualized with FITC-conjugated anti-SAG1.We selected 20 randomly fields of view for each coverslip to determine the percentage of intracellular parasites using confocal laser microscopy at 400×magnification (LSM880+airyscan, ZEISS, German).

### Plaque Assays

Freshly harvested ME49*wt* or ME49*Δcdpk3* tachyzoites (1000 per strain) were added in six-well plates with monolayers of MEF cells. After 10 days of growth at 37°C with 5% CO2, monolayers were fixed and stained with 0.1% crystal violet. Plates were scanned to analyse the number and relative size of plaques. All strains were tested 3 times or more independently, each with three internal replicates.

### Statistical Analysis

Statistical analysis and graphics were performed in GraphPad Prism 8.0 (GraphPad Software Inc., La Jolla, CA, USA). All data were compared by unpaired t-test, one-way analysis of variance. The value of p < 0.05 was considered statistically significant.

## Result

### The ME49*Δcdpk3* Strain Was Constructed Using CRISPR/Cas9 Technology

**Figure 1:**
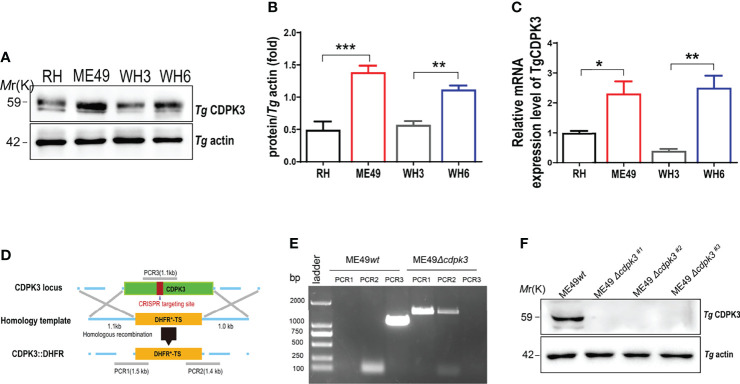
Construction of the ME49*Δcdpk3* strain of *Toxoplasma gondii* by using CRISPR-Cas9 technology. **(A, B)** The expression levels of CDPK3 in four strains of RH, ME49, WH3, and WH6 were detected by Western blotting. *T. gondii* actin was used as a loading control. **(C)**The RNA expression levels of CDPK3 in four strains of RH, ME49, WH3, and WH6 were analyzed by quantitative real-time PCR (RT-qPCR). **(D)** Schematic of CRISPR/CAS9 strategy for replacing CDPK3 with pyrimethamine-resistant DHFR (DHFR*) and PCR identification of single clone. **(E)** PCR identification of ME49*Δcdpk3 T. gondii* strain. PCR1 and PCR2 check for 5’and 3’integration of the selection marker, while PCR3 checks for successful deletion of the CDPK3 gene. Wild-type ME49 (ME49*wt*) strain was used as a control. **(F)** Western blotting for detection of CDPK3 expression in ME49*wt* and ME49*Δcdpk3*. *T. gondii* actin antibody was used as a loading control. Data are mean ± SD of triplicates. Statistical differences are represented by ***p < 0.001, **p < 0.01, *p < 0.05 (n = 3 each group).

*Toxoplasma gondii* CDPK3 gene was identified by comparing and studying the differential gene expression between the strong virulent strains (RH and TgCtwh3 strains) and less virulent strains (ME49 and TgCtwh6 strains). The RT-qPCR and western blot assays showed that *cdpk3* gene was highly expressed in less virulence strains such as ME49 and TgCtwh6 (**Figures 1A–C**). To examine the biological functions of the TgCDPK3 in less virulent strain, CRISPR/Cas9-mediated genome editing was used to knock out CDPK3 in type II ME49 strain. The corresponding CDPK3 targeting CRISPR plasmid pSAG1-Cas9-U6-CDPK3 and homology template CDPK3::DHFR were constructed and electroporated into tachyzoites of the ME49 strain (**Figure 1D**). PCR results proved that DHFR coding sequence was successfully integrated into the CDPK3 locus (**Figure 1E**). Western blotting showed no expression of CDPK3 protein by ME49*Δcdpk3* strain (**Figure 1F**). The result confirmed that the *cdpk3* gene knockout strain of *Toxoplasma gondii* ME49 strain was successfully constructed.

### *Toxoplasma gondii* CDPK3 Activates the Host Autophagy

**Figure 2:**
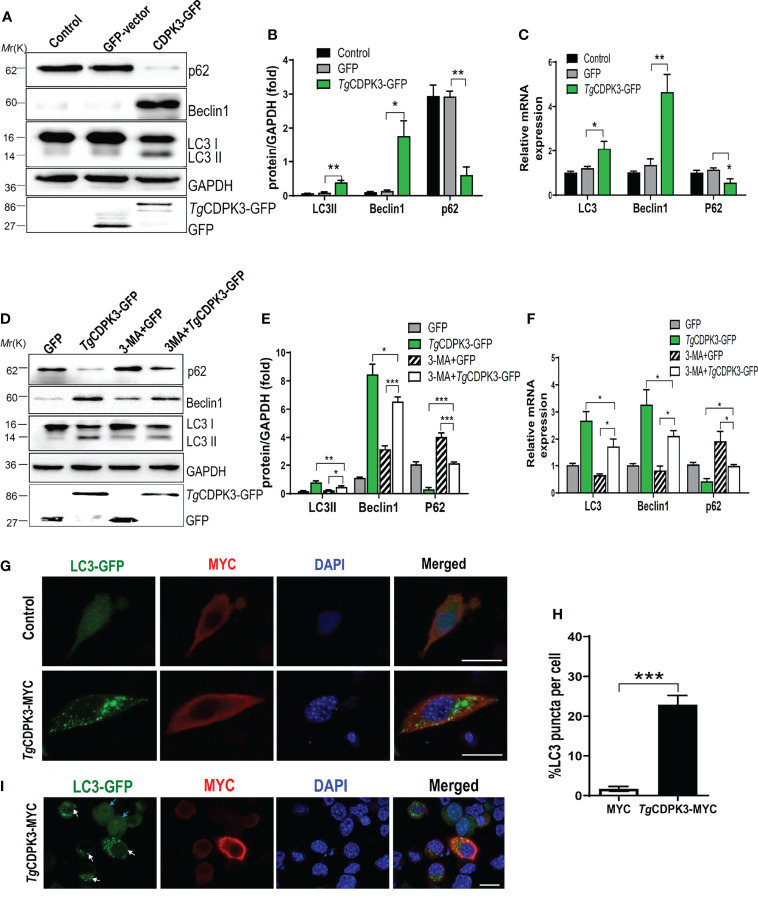
Overexpression of *Tg*CDPK3 enhances autophagy activation. **(A, B)** Western blot analysis of LC3-II, Beclin-1, p62 in cells transfected with *Tg*CDPK3-GFP and control GFP-vector. GAPDH was used as a loading control. **(C)** RT-qPCR analysis of LC3, Beclin-1, p62 RNA levels in cells transfected with *Tg*CDPK3-GFP and control GFP-vector. GAPDH was used as a loading control. **(D, E)** Western blot analysis of autophagy proteins in different groups with pretreatment of a protease inhibitor 3-maleimidobenzoic acid (3-MA;5mM; Sigma-Aldrich, USA). GAPDH was used as a loading control. **(F)** Analysis of autophagic protein RNA levels in different groups with a protease inhibitor 3-MA. GAPDH was used as a loading control. **(G)** Immunofluorescence staining showed LC3 puncta increased significantly in cells transfected with *Tg*CDPK3-MYC. CDPK3-MYC (red), LC3-GFP (green), and DAPI (blue), (Scale bar:10μm). **(H)** Data are mean ± SD of triplicates. Statistical differences are represented by ***p < 0.001, **p < 0.01, *p < 0.05 (n=3 each group). **(I)** Immunofluorescence staining showed LC3 puncta increased significantly in cells transfected with *Tg*CDPK3-MYC. CDPK3-MYC (red), LC3-GFP (green), and DAPI (blue). LC3-GFP puncta in *Tg*CDPK3-MYC transfected cells were noted with white arrows, and LC3-GFP in un-transfected cells were noted with blue arrows (Scale bar:10μm).

To determine whether *T. gondii* CDPK3 affects the host autophagy, CDPK3 labeled with GFP-tagged was transfected into mouse macrophages RAW264.7. Western blotting revealed that overexpression of CDPK3-GFP dramatically induced LC3-I to LC3-II conversion, p62 degradation, and Beclin-1 increase, compared with the control or GFP vector groups (**Figures 2A, B**). RT-qPCR also confirmed that CDPK3-GFP increased the mRNA expressions of LC3 and Beclin-1, while decreased p62 (**Figure 2C**). Consistently, CDPK3 led to a dramatic increase of LC3-II, Beclin-1, and significant decrease of p62, which was relieved by 3-MA (3-methyladenine, an early inhibitor of autophagy) treatment (**Figures 2D–F**). In addition, Immunofluorescence staining showed that CDPK3 overexpression greatly increased the **intensity** of fluorescent LC3 puncta per cell compared to control group (**Figures 2G–I**). These results indicated that CDPK3 activated the host autophagy and significantly increases in **GFP-LC3** puncta.

### ME49*Δcdpk3* Attenuates Autophagy In Murine Macrophage

**Figure 3:**
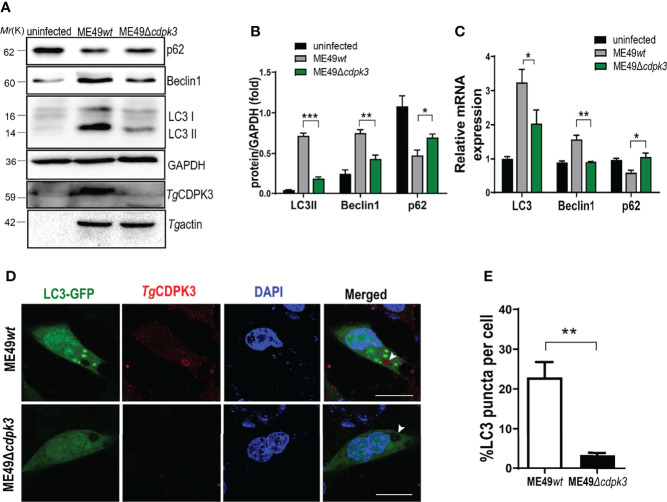
ME49*Δcdpk3* attenuates the activation of autophagy. **(A, B)** ME49*wt* and ME49*Δcdpk3* strain infected RAW264.7 cells for 24 h. Then the cell lysates were analyzed by western blot using indicated antibodies. GAPDH was used as a loading control. **(C)** RAW264.7 cells were infected with ME49*Δcdpk3* and ME49*wt* strains. After 24 hours, the RNA expression level of autophagy status was analyzed by real-time quantitative PCR (RT-qPCR). GAPDH was used as a loading control. **(D, E)** Cells grown on glass coverslips were transfected with LC3-GFP (green) and then infected with ME49*wt* strain and ME49*Δcdpk3* for 24 hours. Double immunofluorescence was performed using anti-CDPK3(red) and DAPI (DNA, blue) (Scale bar:10μm). A distinctive intensity of LC3-GFP was observed in ME49*wt* strain infected cells. Data are mean ± SD of triplicates. Statistical differences are represented by ***p < 0.001, **p < 0.01, *p < 0.05 (n = 3 each group).

To further confirm the relationship between parasitic CDPK3 and the host cell autophagy, mouse macrophages were infected with wild type ME49 (ME49*wt*) and *cdpk3* gene knockout strain ME49(ME49*Δcdpk3*) strains, respectively. After 24 hours post-infection, the expression levels of autophagy proteins were evaluated using western blot and RT-qPCR. The results showed that ME49*Δcdpk3* strain reduced the conversion of autophagy marker LC3-I to LC3-II, downregulated the expression of Beclin-1 and upregulated the expression of p62 compared to ME49*wt* strain, suggesting that CDPK3 activates the host autophagy (**Figures 3A-C**). Immunofluorescence staining showed that ME49*wt* strain greatly increased the intensity of GFP-LC3 compared to ME49*Δcdpk3* strain (**Figures 3D, E**). These data consistently showed that ME49*wt* strain induced the host autophagy compared with ME49*Δcdpk3* parasites. Taken together, we conclude that the host autophagy is activated by CDPK3 of the less virulent ME49 strain during the infection.

### *Toxoplasma gondii* CDPK3 Interacts With Atg3 and Atg5

**Figure 4:**
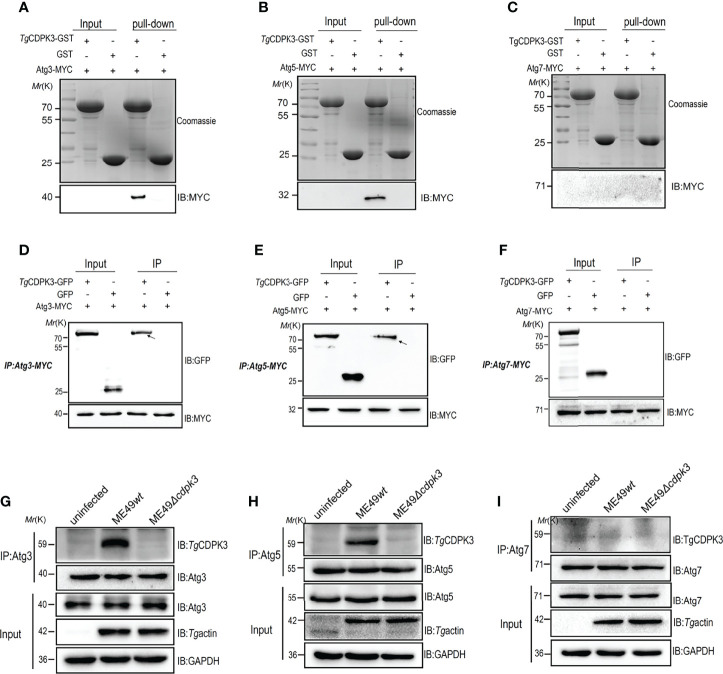
Confirmation of the interaction of *Toxoplasma gondii* CDPK3 with Atg3 and Atg5. **(A–C)** Confirm the binding of CDPK3 to Atg3 and Atg5 *in vitro* pull-down assay. GST-CDPK3 purified on glutathione beads is used as an affinity matrix for Atg3, Atg5 or Atg7. Coomassie brilliant blue staining (upper image), Western blot (MYC tag antibody, lower image) **(D–F)** Verifying that CDPK3 interacts with Atg3 and Atg5 through immunoprecipitation. CDPK3-GFP or control GFP vector with Atg3-MYC, Atg5-MYC, or Atg7-MYC co-transfected into HEK 293T cells. The arrow indicates that CDPK3 (uppe panel) and Atg3-MYC and Atg7-MYC (lower panel) are immunoprecipitated together. **(G–I)** ME49*wt* and ME49*Δcdpk3* strain respectively infected RAW264.7 cells to determine CDPK3 and Atg3 and Atg5 interaction (Moi = 3). At 4 h after infection, rabbit polyclonal CDPK3 antibody was used to detect the immunoprecipitation (IP) of Atg3, Atg5 and Atg7 from the lysate of infected cells.

Previous studies reported that a set of Atg proteins including Atg7, Atg3, and the Atg12-Atg5-Atg16L1 complex are involved in the conjugation of microtubule-associated protein 1 light chain 3 (LC3) to phosphatidylethanolamine (19, 27, 28). To explore the relationship between *Tg* CDPK3 and Atg proteins, we performed an GST pull-down assay to determine the interaction between CDPK3 and autophagy-related proteins Atg3, Atg5 and Atg7. We observed that CDPK3-GST, but not GST, was able to pull down Atg3-myc and Atg5-myc (**Figures 4A, B**). However, CDPK3-GST could not pull down Atg7 (**Figure 4C**). Moreover, immunoprecipitation assay also confirmed that CDPK3 indeed interacts with Atg3 and Atg5 but not Atg7 (**Figures 4D–F**). In order to determine the interaction between parasitic CDPK3 and Atg5 or Atg3 in infected cells, mouse macrophages were infected with ME49*wt* strain or ME49*Δcdpk3* strain about 3 moi, and then polyclonal anti-Atg5, anti-Atg3 antibodies, or anti-Atg7 antibodies were used to immune-precipitate Atg5, Atg3 and Atg7 in the cells. Western blotting showed that CDPK3 from the ME49*wt* parasite was clearly detectable in Atg3, Atg5 immunoprecipitation compared to the ME49*Δcdpk3* parasite (**Figures 4G–I**). The result suggested that the host Atg3 and Atg5 are binding proteins of CDPK3 during infection with the less virulent ME49 strain. Taken together, we conclude that CDPK3 interacts with Atg3 and Atg5.

### *Toxoplasma gondii* CDPK3 Interacts With Host Atg3 and Atg5 to Mediate the Host Autophagy

**Figure 5:**
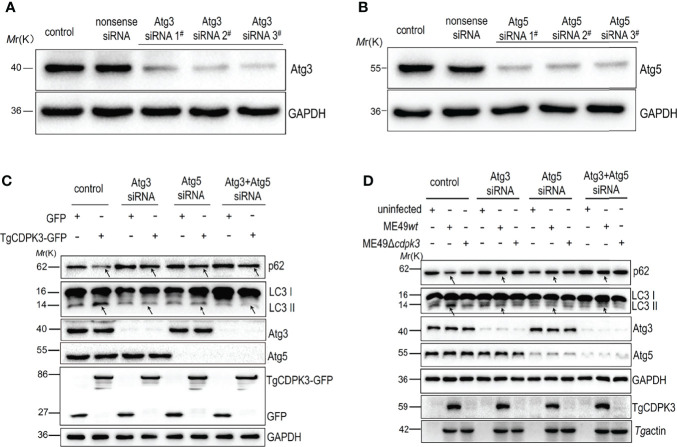
The interference of Atg3 and Atg5 using siRNA down-regulated host cell autophagy induced by *Tg*CDPK3. **(A-B)** Western blot confirmed that Atg3 and Atg5 were successfully interfered. GAPDH was used as a loading control. **(C)** Western blotting analyzed the influence of Atg3 or Atg5 blockage on the expression levels of autophagy proteins LC3II and p62 in CDPK3 overexpressing cells. GAPDH was used as a loading control. **(D)** Western blotting analyzed the influence of Atg3 or Atg5 interference on the expression levels of autophagy proteins LC3II and p62 in cells infected with ME49*wt* and ME49*Δcdpk3* parasites. GAPDH was used as a loading control. *Tg* profilin serves as a control for *Toxoplasma* equivalent loading. The arrow indicates that Atg3 knockdown, Atg5 knockdown, and Atg3 and Atg5 knockdown together significantly inhibited the expression of LC3-II and increased p62 expression.

To further confirm ***Tg*** CDPK3 interaction with host Atg3 and Atg5 to activate autophagy, siRNA was used to downregulate the Atg3 and Atg5 expressions. Western blot analysis results confirmed the efficiency of Atg3 siRNA and Atg5 siRNA (**Figures 5A, B**). As indicated by arrows in **Figure 5C**, Atg3 knockdown, Atg5 knockdown, and both Atg3 and Atg5 knockdown together notably suppressed CDPK3-GFP-induced LC3-I to LC3-II conversion and increased CDPK3-GFP-induced p62 levels. Furthermore, Atg3 knockdown, Atg5 knockdown, and both Atg3 and Atg5 knockdown together notably inhibited ME49*wt* strain induced LC3-I to LC3-II conversion and increased the ME49*wt* strain induced p62 levels (**Figure 5D**, *arrows*). These results indicate that host Atg3 and Atg5 are essential for the host autophagy activated by CDPK3 (**Figures 5C, D**). These results suggested that the host Atg3 and Atg5 are dispensable for the host autophagy induced by *Tg*CDPK3.

### *Toxoplasma gondii* CDPK3 Promotes the Localizations of IFN-γ-Inducible GTPases Around PVM in IFN-γ Induced Murine Macrophages

**Figure 6:**
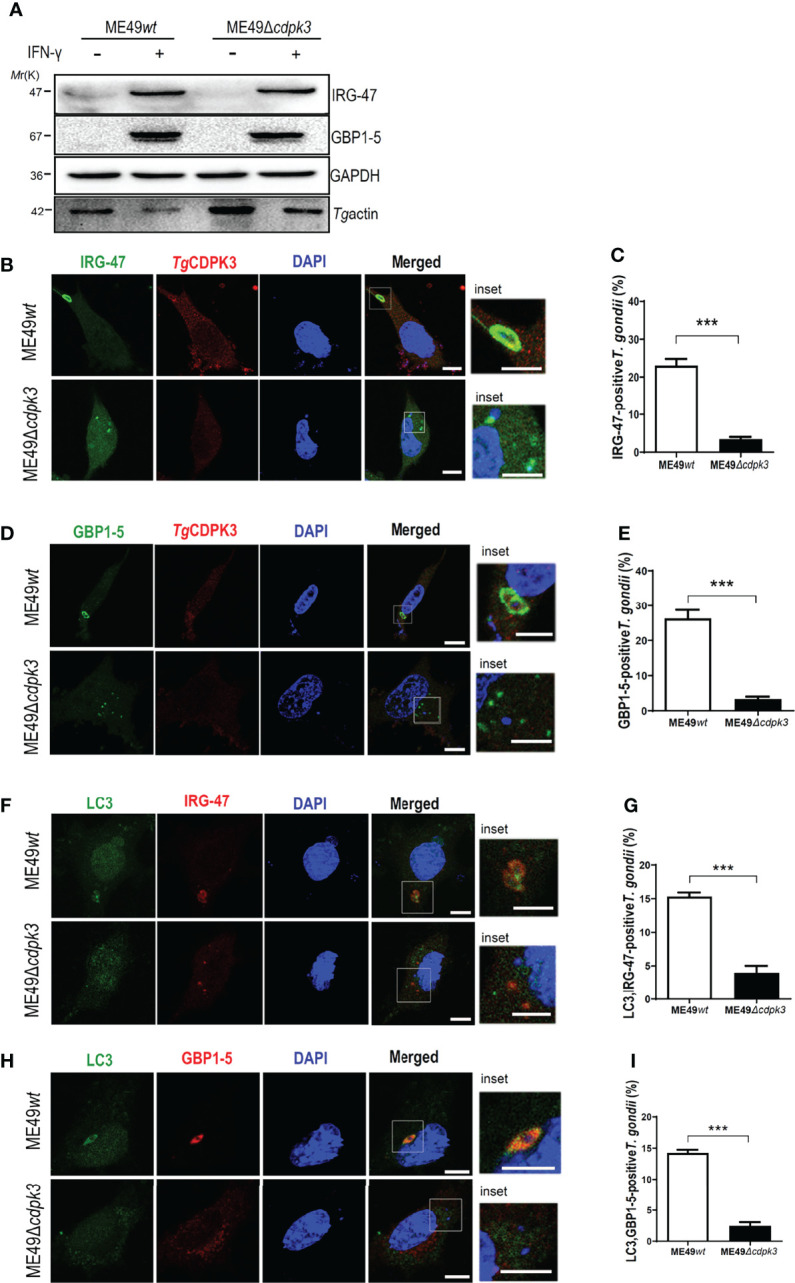
*Tg*CDPK3 plays a critical role in IFN-γ induced IRG-47 and GBP1-5 targeting PV membrane. **(A)** Macrophages (pre-treated with 100 U/ml IFN-γ for 24 h) infected ME49*wt* or ME49*Δcdpk3* parasites were lysed. The lysates were detected by western blot with the indicated antibody. GAPDH was used as a loading control. **(B–E)** Cells (pre -treated with 100U/ml IFN-γ for 24 h) were infected with ME49*wt* or ME49*Δcdpk3* parasites (moi =3), fixed at 4-6 h post-infection, and incubated with rabbit anti-CDPK3 antibody (red), mouse anti-IRG-47 antibody (green) or mouse anti-GBP1-5 antibody (green), and DAPI (blue). (Scale bar:5μm) **(F–I)** Cells (pretreated with 100 U/ml IFN-γ for 24 hours) infected ME49*wt* or ME49*Δcdpk3* parasites (moi =3), fixed at 4-6 h post-infection, and incubated with rabbit anti–LC3II (green), mouse anti-IRG-47(red) or mouse anti-GBP1-5(red), and DAPI (blue). (Scale bar: 5μm) Data are mean ± SD of triplicates. Statistical differences are represented by ***p < 0.001 (n = 3 each group).

**Figure 7:**
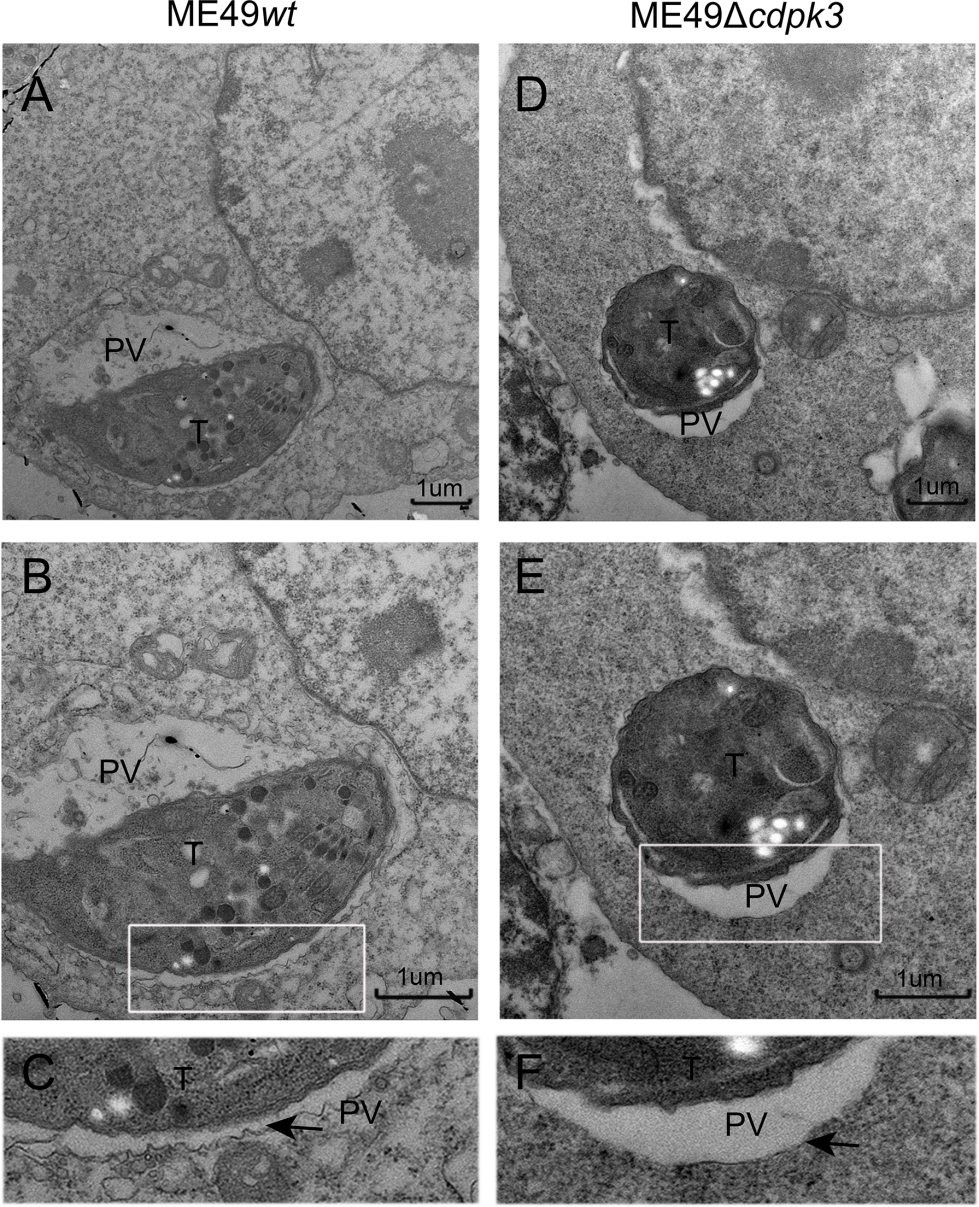
Electron microscopy analysis of the ultrastructural characteristics of parasitic vacuolar membranes in IFN-γ-activated RAW264.7 cells. **(A–D)** Ultrastructural characteristics of parasitic vacuoles in IFN-γ (100 U/ml IFN-γ, 0.1 ng/ml LPS) activated RAW264.7 infected with ME49*wt* or ME49*Δcdpk3* parasites. **(E, F)** A magnification of the PV around parasite in IFN-γ activated RAW264.7 shows the highly scalloped appearance of the membrane (marked with a black arrow). Parasitic vacuoles (PV), *Toxoplasma gondii* (T) (Scale bar:1μm).

Interferon-γ (IFN-γ) has pleiotropic effects on infected cells and results in reduced parasite replication. Much of this IFN-γ-induced host resistance program is dependent on families of IFN-inducible GTPases. The immunity-related GTPases (IRGs) are a family of IFN-γ-inducible GTPases that are essential for host resistance against *Toxoplasma gondii.* The p65 guanylate-binding proteins (GBPs) are another family of enzymes induced by IFN-γ that participate in immunity against the parasite. Atg12-Atg5-Atg16L1 complex, as well as Atg3 and Atg7, which are required for recruitment of IRGs and GBPs to the PV membrane in murine cells, are activated by IFN-γ (20, 29). Without Atg5, IFN-γ induces IRGs and GBPs to form aggregates in the cytoplasm and cannot target to vacuoles containing pathogens (30). Once these IRGs and GBPs were recruited to the LC3-labeled PV membrane, the PV membrane around the parasite showed obvious vesiculation, stripped off the PV membrane of parasites, and restricted the growth of *Toxoplasma gondii* (14, 31). Therefore, we sought to assess whether *Tg*CDPK3-is involved in IFN-γ-dependent cellular immunoregulation in less virulent strain. Although IRG-47 and GBP1-5 expressions in macrophages infected with ME49*Δcdpk3* were as same as the cells infected with ME49*wt* (**Figure 6A**), the percentage of IRG-47 positive vacuoles were significantly low in cells infected with ME49*Δcdpk3* compared to ME49*wt* infected cells (**Figures 6B, C**). Consistently, the localizations of GBP1-5 on the parasitophorous vacuole membrane was significantly reduced in cells infected with ME49*Δcdpk3* strain, and they formed aggregates in the cytoplasm (**Figures 6D, E**). As mentioned above, *Tg*CDPK3 activates the host autophagy and significantly increases GFP-LC3 puncta (**Figures 2G–I**). Previous studies have found a discrepancy in cell localization and function between N-terminal GFP-tagged LC3 and endogenous LC3 (18, 20, 32). Therefore, we further determined the localization of endogenous LC3 and IFN-γ-induced effectors IRG-47 and GBP1-5. Immunofluorescence results showed that the endogenous LC3 were co-localized with IRG-47 and GBP1-5 on the PVM of the parasites (**Figures 6F, H**). The percentage of LC3 co-localized with IRG-47 or GBP1-5 on the PV membrane of ME49*Δcdpk3* parasites was significantly decreased compared with ME49*wt* strain (**Figures 6G, I**). Furthermore, electron microscopy of IFN-γ-activated macrophages showed that CDPK3-deficient parasites were found in more a closely-fitting vacuole which had a smooth circumference, characteristic of an intact PV. Instead, ME49*wt* parasites showed obvious vesiculation and rupture of the vacuole membrane, indicating the disruption of PV membrane of wild type parasites by these IFN-γ inducible immunity-related proteins (**Figure 7**). Taken together, we conclude that *Tg*CDPK3 promotes the localizations of IRG-47 and GBP1-5 around PV to destroy the vacuole membrane.

### *Toxoplasma gondii* CDPK3 Restricts Parasitic Proliferation in Murine Macrophages

**Figure 8:**
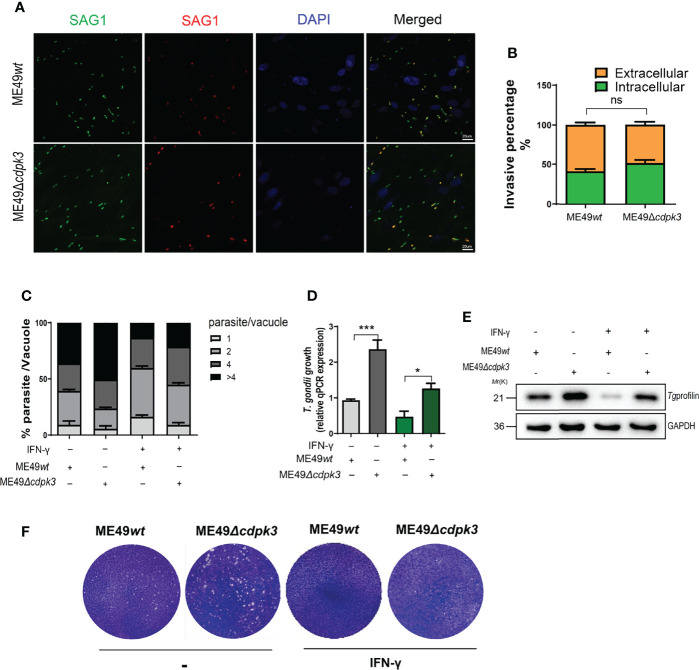
*Tg*CDPK3 affects parasite growth *in vitro*. **(A, B)** ME49*wt* or ME49*Δcdpk3* parasites were allowed to invade confluent MEF cells for 2 hours. (Scale bar:20 μm). Determine the percentage of intracellular parasites. **(C-E)** MEF cell was stimulated with 100 U/mL IFN-γ for 24 h. Following stimulation, equal mount ME49*wt* or ME49*Δcdpk3* parasites infected MEF cells for 24 hours. **(C)** A total of at least 200 vacuoles were inspected to determine the number of vacuoles containing 2, 4, 8, or 16 parasites. **(D)**Parasite growth was also quantified by qPCR of genomic DNA using the SAG1 primers and normalized against β-actin. **(E)**Total infected cell proteins at 24 hours after *Toxoplasma* infection were analyzed by immunoblotting to detect *T. gondii* profilin protein. GAPDH was used as a loading control. **(F)** Plaque assay comparing the growth of ME49*wt* or ME49*Δcdpk3* parasites. Data are mean ± SD of triplicates. Statistical differences are represented by ***p < 0.001, *p < 0.05, ns, no significant (n=3 each group).

Given the correlation between *Toxoplasma gondii* CDPK3 and IFN-γ inducible immunity-related proteins accumulation on PV membrane, we hypothesized that *Tg*CDPK3-might contribute to IFN-γ-induced cell-autonomous resistance to the parasites. First, we compared the invasion and proliferation abilities of ME49*wt* strain and ME49*Δcdpk3* strain in MEF cells. The result suggested that CDPK3 has no significant effect on parasite invasion (**Figure 8A, B**), which is consistent with the previous study (33). Next, we determined whether or not *Tg*CDPK3-affects intercellular parasites growth. The result showed that 4 or more parasites accounted for a higher proportion in the vacuoles of ME49*Δcdpk3* strain, suggesting that *Tg*CDPK3-inhibits the intracellular proliferation of parasites (**Figure 8C**). Consistently, IFN-γ stimulation resulted in a larger loss in the number of parasites per vacuole, and there was a significant decrease in MEF cells infected with ME49*wt* parasites compared with ME49*Δcdpk* parasites (**Figures 8C, D, E**). Moreover, plaque assays were performed on the wild type parasites and CDPK3-deficient parasites to test whether CDPK3 caused a growth defect. At 10 days post-infection, the size of plaques and the number of plaques per well were calculated. The result showed that IFN-γ stimulation resulted in a decrease in the number and size of plaques formed, and the number and size of ME49*Δcdpk3* strain plaques was larger than that of the ME49*wt* strain (**Figure 8F**). Taken together, all of these results confirmed that *Tg*CDPK3 would inhibit the intracellular proliferation of parasites.

## Discussion

T. gondii can invade almost all nucleated cell types and establishes a chronic infection in hosts by forming cysts in various sites such as brain, cardiac and skeletal muscles (34). To establish successful parasitism, *T. gondii* has developed several pathways to ensure invasion and exit from cells, and therefore cell-intrinsic host defense is crucial for limiting parasite proliferation and cyst formation (35). CPDKs have been identified in plants and protozoans (36, 37), and are known to play crucial roles in the signal transduction pathways. *Tg*CDPK3 has been implicated in controlling calcium-dependent permeabilization of the PV membrane and regulate parasite egress from the host cell, and plays an important role in the egress of the parasite (23, 38). *Tg*CDPK3 is located on the periphery of the parasite, and its function in cellular immunity has rarely been studied (24, 25).

The best-studied *Toxoplasma gondii* -host cell relationship is that between virulent tachyzoites and non-phagocytic, fibroblastic mammalian cell lines. This relationship begins with the virulent tachyzoite actively invades host cells by forming a moving junction (MJ) at the host cell membrane. During invasion, the host cell’s plasma membrane is invaginated, ultimately completely encompassing the tachyzoites in a parasitophorous vacuole (PV). However, unlike virulent *T. gondii* strains that actively invade host cells at the plasma membrane, their less virulent counterparts mainly use the phagosome to vacuole invasion (PTVI) pathway–a noncanonical infection pathway as the invasion into macrophages through phagocytosis and subsequent active penetration from within the phagosome to form a PV. During this process, the surface effectors of the less virulent tachyzoites may have the chance to interact with the host cytoplasmic proteins to regulate cellular immunity (24, 25). Based on this characteristic of the less virulent strain, we studied the immunoregulation of macrophages by *Tg*CDPK3. In this present study, we demonstrated that *Tg*CDPK3 interacts with autophagy related proteins Atg3 and Atg5 to recruit immune-related proteins IRGs and GBPs on PV membrane during the infection with macrophage of the type II strain, which is beneficial to the less virulent strain of *T. gondii* long-term latency in host cells. Previous studies demonstrated that *T. gondii* infection induces host cell autophagy (39–42). Our study revealed that *Tg*CDPK3 induced autophagy in murine macrophages. Consistently, CDPK3-deficient parasites not only significantly reduced expression levels of LC3 II and Beclin1, but also significantly increased the expression of p62 as a result of impaired or defective autophagy. Consistently, *Tg*CDPK3 overexpression led to a significant increase of LC3-II, Beclin-1, and decrease ed p62 which was prevented by 3-MA treatment.

IFN-γ-mediated autophagy pathway which normally is involved in cellular remodeling and nutrient recycling, also plays crucial roles in host defense (43). The components of the Atg pathway that mediate the processing, lipidation and recruitment of LC3 to the PV membrane are required for control of *T. gondii* infection in IFN-γ-stimulated murine and human cells (44). The key Atg proteins involved in this pathway include Atg7, Atg3, and Atg12-Atg5-Atg16L1 complex, are needed for the PV membrane recruitment of IRGs and GBPs, and vesiculation of the PV in IFN-γ-stimulated murine cells (20, 30, 45). Previous studies have demonstrated that three *Toxoplasma* effector molecules, ROP18, ROP5, and GRA15 that determine strain-specific accumulation of IFN-γ-inducible GTPases on the PV. These highly polymorphic proteins are held responsible for a large part of the strain-specific differences in virulence. Type I and type II ROP18 can phosphorylate IRGs and GBPs to destroy their accumulation on PVM and avoid clearance in murine cells (12, 46), whereas less virulent type II and avirulent III strains have the highest percentage of IRGs-coated vacuoles (13, 47). The reason is that the absence of the virulence allele of pseudokinase ROP5 in type II strains accounts for the inability of ROP18 to disrupt IRGs recruitment on the PVM (46, 48). In addition, type II GRA15 interacts with TRAF2 and TRAF6, and TRAF6 recruitment enhances parasite susceptibility to IRGs/GBPs-dependent elimination in MEFs (49).

Herein, we report that *Tg*CDPK3 interacts with Atg3 and Atg5, which results in the recruitment of IRGs and GBPs around the parasitic PV, and LC3 and GBP1-5, IRG-47 co-localization at PV membrane. IFN-γ-activated murine macrophages showed marked vacuole vesiculation and disruption of ME49*wt* parasites, whereas CDPK3-deficient parasites were found in more a closely fitting vacuole which had a smooth circumference, characteristic of an intact PV membrane (**Figure 7**), indicating that CDPK3 of less virulent strains of *T. gondii* can promote autophagic targeting and killing of the parasite in PV. Therefore, CDPK3 interacts with host Atg3 and Atg5 to drive LC3 lipidation and autophagosome production. Moreover, CDPK3 is involved in the recruitment of LC3-II and immunomodulatory proteins to the PVM in IFN-γ-stimulated cells and it is important for disruption of the PVM, reduced parasite replication and viability. Compared to low CDPK3 expression in virulent strains, the increased expression of CDPK3 in less virulent could reduce virulence in these strains through an improved IFN-γ-mediated intracellular defense against “CDPK3-high” parasites. Furthermore, to enhance its chances of transmission, the less virulent parasites needs to reduce parasite burdens in the host cell to prevent killing its host, thus to balance immune evasion, to enable replication and dissemination before tissue cysts are formed.

It has been widely accepted that *Toxoplasma gondii* type II, that is less virulent strain, is the most prevalent genotype in the world, however, the underlying mechanisms remains elusive. In the light of our results, we consider that *Tg*CDPK3 interacts with autophagy-related proteins, promoting cell-autonomous immunity to restrict the parasitic replication, which is beneficial to establish long-term latency and resist infection of less virulent strains.

## Data Availability Statement

The original contributions presented in the study are included in the article/**Supplementary Material**. Further inquiries can be directed to the corresponding authors.

## Ethics Statement

The animal study was reviewed and approved by the Institutional Review Board of the Institute of Biomedicine at Anhui Medical University.

## Author Contributions

JD and LC, conceived and designed the experiments, writing - review and editing. MW, RA, and NZ, performed the experiments, methodology, and formal analysis. YC, HC, QY, and RW, methodology and formal analysis. QL and LY, formal analysis. All authors contributed to the article and approved the submitted version.

## Funding

This work was supported by the National Natural Science Foundation of China (No. 82072300, No. 81871674, and No. 81902084).

## Conflict of Interest

The authors declare that the research was conducted in the absence of any commercial or financial relationships that could be construed as a potential conflict of interest.

## Publisher’s Note

All claims expressed in this article are solely those of the authors and do not necessarily represent those of their affiliated organizations, or those of the publisher, the editors and the reviewers. Any product that may be evaluated in this article, or claim that may be made by its manufacturer, is not guaranteed or endorsed by the publisher.
