# Supplementary material for: Toxoplasma gondii CDPK3 Controls the Intracellular Proliferation of Parasites in Macrophages
Source: Front Immunol. 2022 Jun 10;13:905142. doi: 10.3389/fimmu.2022.905142 (PMC9226670; doi:10.3389/fimmu.2022.905142)
Supplement: Supplementary file 1 [file Table_1.docx]

| **Primers** | **Sequence** | **Used for** |
| --- | --- | --- |
| CDPK3-guide RNA (gRNA)-F | TGTACCGAGGGTTTTAGAGCTAGAAATAGC | Q5 mutagenesis changing the gRNA in pSAG1:CAS9-U6:sgUPRT to gRNA-CDPK3 |
| CDPK3-gRNA-R | CCTCCATGACAACTTGACATCCCCATTTAC |  |
| UpCDPK3-F | AAAACGACGGCCAGTGAATTC AGCCAACATGCATTGGAGCT | To produce UpCDPK3 PCR product for making pCDPK3: DHFR |
| UpCDPK3-R | GGGGGTGAAAATCGAATGACA ACGCAGCGACTGGGAGAATC |  |
| DHFR-TS-F | TGTCATTCGATTTTCACCCCC | To produce DHFR PCR product for making pCDPK3: DHFR |
| DHFR-TS-R | AGTGTGATGACTCCGCAACT GGATCGATCCCCCCGGGCTGC |  |
| DnCDPK3-F | GCAGCCCGGGGGGATCGATCC AGTTGCGGAGTCATCACACT | To produce DnCDPK3 PCR product for making pCDPK3: DHFR |
| DnCDPK3-R | GACCATGATTACGCCAAGCTT TCGTTGTGGGCTATACAGCT |  |
| PCR1-F | GCCTAACAAGGATTCGATCAGTAGC | To examine the integration of DHFR-TS into corresponding genes |
| PCR1-R | TGTCGTGGATTTACCAGTCATGGAC |  |
| PCR2-F  PCR2-R | TGACTCTTCATGTGGCATTTCACAC  TACTGTGTTAGGTAGCAAATGTGG | To examine the integration of DHFR-TS into corresponding genes |
| PCR3-F | TCGTGCGTCTTCAGGCATGTACATC | To examine the deletion of CDPK3 sequences |
| PCR3-R | GAGGTCCTTTCGCTTCCTGAGACTC |  |

**Supplementary table 1: Primers for construction of CDPK3-deficient ME49 strain.**

**.**
