# Supplementary material for: Toxoplasma gondii CDPK3 Controls the Intracellular Proliferation of Parasites in Macrophages
Source: Front Immunol. 2022 Jun 10;13:905142. doi: 10.3389/fimmu.2022.905142 (PMC9226670; doi:10.3389/fimmu.2022.905142)
Supplement: Supplementary file 2 [file Table_2.docx]

| **Primers** | **Forward primer (5′–3′)** | **Reverse primer (5′–3′)** |
| --- | --- | --- |
| LC3 | TTATAGAGCGATACAAGGGGGAG | CGCCGTCTGATTATCTTGATGAG |
| Beclin1 | ATGGAGGGGTCTAAGGCGTC | TGGGCTGTGGTAAGTAATGGA |
| p62 | ACAGCCCAAACGTGCAGTAA | CTGATGCGGAACTACATCTGAAT |
| GAPDH | AGGTCGGTGTGAACGGATTTG | GGGGTCGTTGATGGCAACA |
| *Tg*CDPK3 | GGACTCAAGTGTCGGACGAG | TCTGGGAGCAGAACTTGACG |
| *Tg*SAG1 | GTGCCACGCTAACGATCAAG | TGGAAACGTGACTGGCTGTT |
| β-actin | AGCTTCTTTGCAGCTCCTTCGT | TACACGCTAGGCGTAAAGTTGG |

**Supplementary table 2:** **The primers used for qRT-PCR.**
